# Enhanced and Selective Antiproliferative Activity of Methotrexate-Functionalized-Nanocapsules to Human Breast Cancer Cells (MCF-7)

**DOI:** 10.3390/nano8010024

**Published:** 2018-01-04

**Authors:** Catiúscia P. de Oliveira, Sabrina L. Büttenbender, Willian A. Prado, Aline Beckenkamp, Ana C. Asbahr, Andréia Buffon, Silvia S. Guterres, Adriana R. Pohlmann

**Affiliations:** 1 Programa de Pós-Graduação em Ciências Farmacêuticas, Faculdade de Farmácia, Universidade Federal do Rio Grande do Sul, Porto Alegre 90610-000, RS, Brazil; catipadilha@yahoo.com.br (C.P.d.O.); alinee-b@hotmail.com (A.B.); andreia.buffon@ufrgs.br (A.B.); silvia.guterres@ufrgs.br (S.S.G.); 2 Programa de Pós-Graduação em Química, Departamento de Química Orgânica, Instituto de Química, Universidade Federal do Rio Grande do Sul, Porto Alegre 91501-970, RS, Brazil; sbuttenbender@gmail.com (S.L.B.); will_ap20@hotmail.com (W.A.P.); 3 Programa de Pós-Graduação em Nanotecnologia Farmacêutica, Universidade Federal do Rio Grande do Sul, Porto Alegre 90610-000, RS, Brazil; acasbahr@hotmail.com

**Keywords:** methotrexate, surface functionalized lipid-core nanocapsules, breast cancer, cellular uptake, antiproliferative activity

## Abstract

Methotrexate is a folic acid antagonist and its incorporation into nanoformulations is a promising strategy to increase the drug antiproliferative effect on human breast cancer cells by overexpressing folate receptors. To evaluate the efficiency and selectivity of nanoformulations containing methotrexate and its diethyl ester derivative, using two mechanisms of drug incorporation (encapsulation and surface functionalization) in the in vitro cellular uptake and antiproliferative activity in non-tumoral immortalized human keratinocytes (HaCaT) and in human breast carcinoma cells (MCF-7). Methotrexate and its diethyl ester derivative were incorporated into multiwall lipid-core nanocapsules with hydrodynamic diameters lower than 160 nm and higher drug incorporation efficiency. The nanoformulations were applied to semiconfluent HaCaT or MCF-7 cells. After 24 h, the nanocapsules were internalized into HaCaT and MCF-7 cells; however, no significant difference was observed between the nanoformulations in HaCaT (low expression of folate receptors), while they showed significantly higher cellular uptakes than the blank-nanoformulation in MCF-7, which was the highest uptakes observed for the drug functionalized-nanocapsules. No antiproliferative activity was observed in HaCaT culture, whereas drug-containing nanoformulations showed antiproliferative activity against MCF-7 cells. The effect was higher for drug-surface functionalized nanocapsules. In conclusion, methotrexate-functionalized-nanocapsules showed enhanced and selective antiproliferative activity to human breast cancer cells (MCF-7) being promising products for further in vivo pre-clinical evaluations.

## 1. Introduction

Nanocarrier drug delivery systems are very promising strategies for the treatment of tumors leading to improved therapeutic efficacy [1,2,3] and reduced drug doses [4,5]. The incorporation of drugs into nanocarriers can be performed by either encapsulation or surface functionalization [6,7,8]. Drug-loaded nanoparticles accumulate at the target tissues or organs due to diverse factors, such as permeability of barriers, tissue damage and pH environment [9,10]. Active cellular targeting can be achieved by functionalizing the surface of nanoparticles with ligands that promote cell-specific recognition and binding, increasing the treatment specificity [11,12,13]. Among the targets, there is the folate receptor [13,14], which is present in the cell membrane and participates in the process of cell replication [15]. Tumor cells replicate more rapidly than normal cells expressing high level of folate receptors. In this sense, the functionalization of nanoparticles with folic acid, or its antagonists, constitutes a promising strategy to improve the efficacy and the selectivity of breast cancer treatment [16,17,18].

Methotrexate is a folic acid antagonist and acts as a ligand for the folate receptor. The incorporation of this drug into nanocarrier systems is an interesting approach, since it increases the drug antiproliferative action [18], improves the specificity for the organ or target tissue [19] and increases the anti-inflammatory action [20]. Additionally, the cellular uptake of magnetic nanoparticles [21] conjugated with methotrexate has improved in either 10-times (HeLa) or 20-times (MCF-7), compared to cells with low expression of folate receptor, such as cardiomyocytes, demonstrating higher specificity. However, as methotrexate is a drug with low lipophilicity, encapsulation in organic and biodegradable nanoparticles becomes difficult. Some alternatives are either the derivatization of the molecule, making it more lipophilic without losing the biological activity [22] or its use as ligand to functionalize the surface of the nanocarrier systems, as we have previously proposed for an antibody fragment [23]. Functionalization is based on the formation of an organometallic complex on the nanoparticle surface, using chitosan-coated lipid-core nanocapsules as nanocarriers, zinc (II), as metal ion and a molecule containing oxygen and/or nitrogen atoms, as ligand. The transition metal ion forms a chelate with chitosan and ligand on the nanoparticle surface [24]. We hypothesized that methotrexate, which contains amino acid functional groups, could be an effective ligand to functionalize the surface of those nanocapsules. Furthermore, this molecule could be incorporated into the nanocapsules by two mechanisms: encapsulation or surface functionalization, influencing the antiproliferative action of nanoformulations.

Breast cancer is the most common cancer in women [25,26]. Advances in cancer treatment have been improving the survival rates of patients. However, cancer is a heterogeneous disease [27] and the treatment involves multiple drugs that may cause many adverse effects [28,29]. Therefore, cancer cells can develop resistance to drugs and methotrexate resistance has been well documented [30,31]. Therefore, it is necessary to develop alternative drug delivery systems in order to improve therapeutic efficacy. Recently, we have showed that methotrexate-diethyl ester-loaded lipid-core nanocapsules increased antineoplastic effects on resistant breast cancer cell line [22].

**Figure 1:**
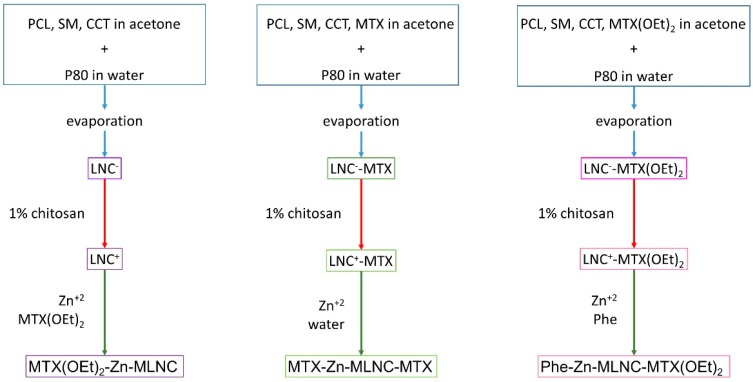
Scheme of synthesis: lecithin-polysorbate 80-coated lipid-core nanocapsules (LNC^−^) containing or not drug, methotrexate (MTX) or methotrexate diethyl ester [MTX(OEt)_2_], prepared using poly(ε-caprolactone) (PCL), sorbitan monostearate (SM) and capric-caprylic triglyceride (CCT), were reacted with 1% chitosan producing chitosan-lecithin-polysorbate 80-coated lipid-core nanocapsules [LNC^+^, LNC^+^-MTX or LNC^+^-MTX(OEt)_2_], which were used to obtain multiwall lipid-core nanocapsules (MLNC) complexed with Zn^2+^ and (i) methotrexate diethyl ester [MTX(OEt)_2_-Zn-MLNC]; (ii) encapsulated and functionalized methotrexate (MTX-Zn-MLNC-MTX); and (iii) methotrexate diethyl ester-encapsulated in the multiwall lipid-core nanocapsules which surface is passivate with phenylalanine [Phe-Zn-MLNC-MTX(OEt)_2_].

Thus, an anti-tumor activity could be improved by functionalizing the surface of multiwall lipid-core nanocapsules with methotrexate taking advantage of its ability to interact with the folate receptor and to act as an antiproliferative agent. Considering the physicochemical characteristics of methotrexate, our first step was to synthesize three different multiwall lipid-core nanocapsules (MLNC). The first one was prepared using methotrexate incorporated by encapsulation and by surface functionalization (organometallic complex) (MTX-Zn-MLNC-MTX); the other two formulations were prepared using methotrexate diethyl ester, a more lipophilic drug derivative, by encapsulation into the nanocapsules [Phe-Zn-MLNC-MTX(OEt)_2_] or by surface functionalization [MTX(OEt)_2_-Zn-MLNC] (Figure 1). The formulations were evaluated considering their cellular uptake and in vitro antiproliferative activity in non-tumoral immortalized human keratinocytes (HaCaT) with low expression of folate receptors and in human breast carcinoma cells (MCF-7), which overexpress folate receptors.

## 2. Results

### 2.1. Synthesis of Functionalized-Zn-MLNC Formulations 

**Figure 2:**
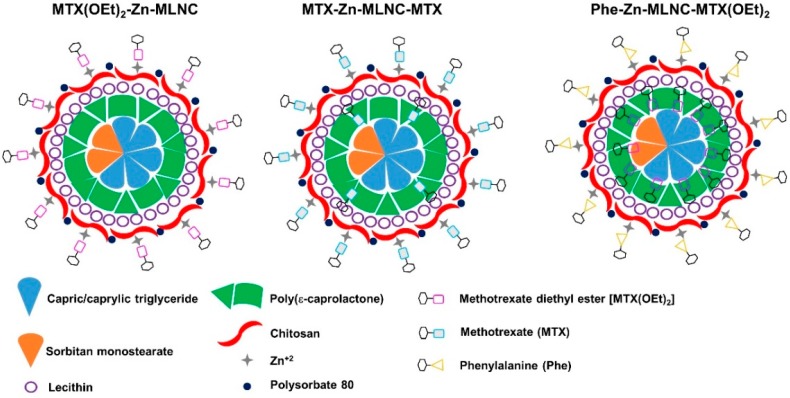
Illustrative models for the supramolecular structures of MTX(OEt)_2_-Zn-MLNC, MTX-Zn-MLNC-MTX and Phe-Zn-MLNC-MTX(OEt)_2_.

The nanoformulations were prepared containing MTX or MTX(OEt)_2_, as described above. Considering the experimental procedures to obtain the nanoformulations, as well as the lipophilicity of MTX or MTX(OEt)_2_ in the LNC-environment (log D of −2.4 and 2.2, respectively), we proposed the supramolecular structural models for each nanocapsule (Figure 2). MTX(OEt)_2_-Zn-MLNC was obtained from drug-unloaded lipid-core nanocapsules coated with lecithin-chitosan-polysorbate 80 and its surface was reacted with Zn^2+^ and methotrexate diethyl ester. MTX-Zn-MLNC-MTX was formed from a dispersion of nanocapsules partially encapsulating MTX and its surface was coated with lecithin-chitosan-polysorbate 80. The LNC^+^-MTX was reacted with Zn^2+^, which caused the complexation of methotrexate initially soluble in the continuous phase of LNC-MTX. The third formulation, Phe-Zn-MLNC-MTX(OEt)_2_ was obtained from MTX(OEt)_2_-loaded lipid-core nanocapsules coated with lecithin-chitosan-polysorbate 80, which surface is reacted with Zn^2+^ and phenylalanine. The formulations were prepared in triplicate. Intermediate product, lipid-core nanocapsules coated with chitosan-lecithin-polysorbate 80 (LNC^+^) and a second blank-formulation (Phe-Zn-MLNC) were also prepared as control groups in cell viability studies.

Blank-nanoformulations (LNC^+^ and Phe-Zn-MLNC), prepared without drug, showed homogeneous white opalescent macroscopic aspect, while the nanoformulations prepared with MTX and MTX(OEt)_2_ [Phe-Zn-MLNC-MTX(OEt)_2_, MTX(OEt)_2_-Zn-MLNC and MTX-Zn-MLNC-MTX] had yellowish opalescent macroscopic aspect (data not shown). In parallel, rhodamine B-labeled formulations were prepared using PCL-RhoB conjugate blended with PCL [**f**-LNC^+^, **f**-Phe-Zn-MLNC-MTX(OEt)_2_, **f**-MTX(OEt)_2_-Zn-MLNC and **f**-MTX-Zn-MLNC-MTX], exhibiting macroscopic homogenous pink whitish aspect (data not shown). The formulations presented pH values (without dilution) between 3.60 ± 0.08 and 4.24 ± 0.15 due to the presence of acetic acid used to disperse chitosan in aqueous medium. One batch of a double-fluorescent-labeled formulation (**ff**-MTX-Zn-MLNC-MTX) was prepared using PCL-RhoB, a polymer conjugate and 5AHBO-C8, a highly lipophilic benzoxazole dye derivative. PCL-RhoB was blended with PCL at the polymer wall and 5AHBO-C8 was dispersed with sorbitan monostearate in MCT oil. This strategy was used to investigate the co-localization of both lipophilic domains of the nanocapsules (lipid-core and polyester wall) in the cellular uptake study. Laser diffraction analysis was used to determine the characteristic size distribution curves and to investigate if the formulations were contaminated by microaggregates or microparticles. All size distribution profiles presented exclusively submicrometric particle populations. Blank-nanoformulations (LNC^+^ and Phe-Zn-MLNC) and drug-contained nanoformulations [Phe-Zn-MLNC-MTX(OEt)_2_, MTX(OEt)_2_-Zn-MLNC and MTX-Zn-MLNC-MTX] had unimodal diameter distribution showing volume-weighted mean diameters (D[4,3]) smaller than 135 ± 10 nm, with polydispersity (SPAN) below 1.1 ± 0.1. The specific surface area (SA) of nanocapsules ranged from 52 ± 1 to 56 ± 5 m^2^·g^−1^ (Table 1). Regarding the fluorescent-labeled formulations, the laser diffraction analysis showed D[4,3] smaller than 190 nm (Table 2).

Taking into account that the formulations did not show any microscopic contaminants and had exclusively size distribution profiles below 1 mm, the dynamic light scattering (DLS) and the nanoparticle tracking analysis (NTA) were used. Those methods provided the size distribution profiles of samples based on the translational diffusion coefficient (Brownian motion of particles suspended in a fluid). All samples showed unimodal size distributions by DLS. The hydrodynamic diameters (D*_z-ave_*), calculated using the method of Cumulants, ranged from 144 ± 9 to 160 ± 32 nm with polydispersity indexes (PDI) below 0.16 (Table 1 and Table 2). The zeta potential varied from +14 ± 3 to +20 ± 6 mV (Table 1 and Table 2). Using NTA, the hydrodynamic diameter (D*_h_*) varied from 140 ± 9 to 176 ± 13 nm for those nanoformulations. The diameter at percentile 90 under the size distribution curve ranged from 190 ± 3 to 243 ± 11 nm and the particle number density ranged from (4.7 ± 0.5) × 10^12^ to (5.7 ± 0.1) × 10^12^ particles mL^−1^ (Table 1).

The experimental concentration of zinc-II in the blank-formulation (Phe-Zn-MLNC) and in the drug-contained formulations (fluorescent-labeled or not) ranged from 76 to 100 mg·mL^−1^ (Table 1 and Table 2). Furthermore, the drug contents [concentrations of MTX or MXT(OEt)_2_] varied from 101 ± 3 µg·mL^−1^ to 115 ± 4 µg·mL^−1^, with encapsulation efficiency higher than 94% (Table 1 and Table 2).

### 2.2. Cellular Uptake Studies

**Figure 3:**
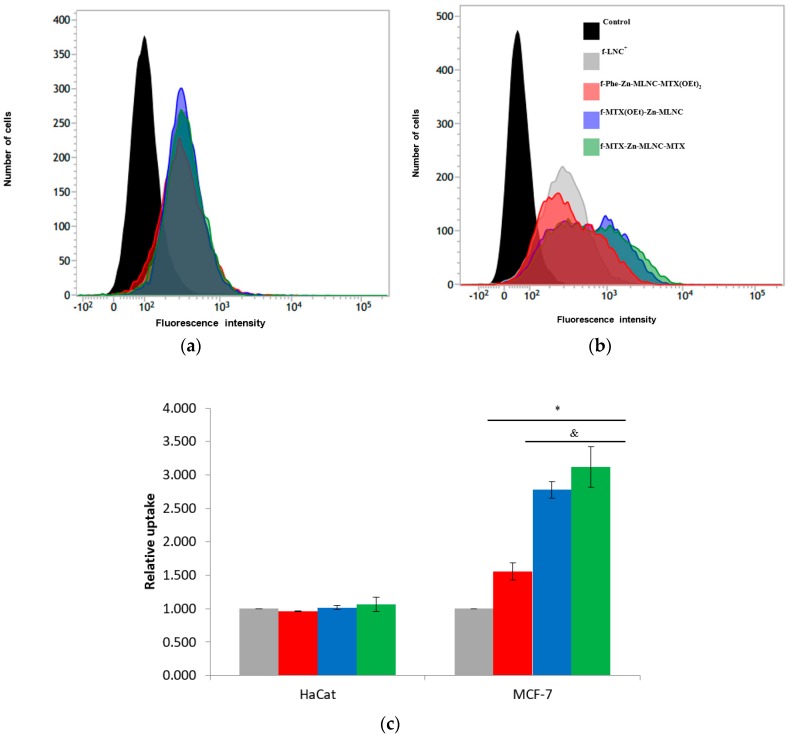
Flow cytometry plot showing the fluorescence intensity versus the number of cells for (**a**) HaCaT (spontaneously immortalized human epithelial cell line) and (**b**) MCF-7 (human breast carcinoma cells), in which the nanoformulations (color distributions) are compared (*p* < 0.05) to the control (untreated cells, black distribution); (**c**) Histogram of the relative cellular uptake for **f**-LNC^+^ (100%), **f**-Phe-Zn-MLNC-MTX(OEt)_2_, **f**-MTX(OEt)_2_-Zn-MLNC, **f**-MTX-Zn-MLNC-MTX. Data represent average ± SD (*n* = 3). No significant difference among formulations (*p* > 0.05) was determined for cellular uptake (ANOVA, Tukey). For MCF-7 cells, Phe, MTX and MTX(OEt)_2_-surface functionalized nanoformulations showed higher nanocapsule cellular uptake than **f**-LNC^+^ (* *p* < 0.05; ANOVA, Tukey). MTX and MTX(OEt)_2_-surface functionalized nanoformulations showed higher nanocapsule cellular uptake than **f**-Phe-Zn-MLNC-MTX(OEt)_2_ (& *p* < 0.05; ANOVA, Tukey).

The spontaneously immortalized human epithelial cell line (HaCaT) was studied as a model for the in vitro nanoparticle uptake by healthy cells (non-tumorigenic control cells). The volume of formulations applied to each well corresponds to a surface area of nanocapsule of 12 cm^2^·mL^−1^ and to a concentration of MTX or MXT(OEt)_2_ of 0.17 mmol·L^−1^. The increase in the fluorescence intensity (*p* < 0.05) observed for the treatment groups compared to the control (untreated HaCaT cells) demonstrated the internalization of the nanocapsules (Figure 3a). The maximum fluorescence intensity determined for the group receiving **f**-LNC^+^ was used as reference of the nanocapsules uptake by the HaCaT cells. The maximum fluorescence intensity observed for the nanoformulations containing MTX and MTX(OEt)_2_ were similar to them and to the reference (**f**-LNC^+^) (*p* > 0.05) (Figure 3c).

The cellular uptake study was also conducted using human breast carcinoma cells (MCF-7). Similar doses were applied to the cell culture (surface area of nanocapsule applied was 12 cm^2^·mL^−1^; and MTX or its diethyl ester concentration was 0.17 mmol·L^−1^). After 24 h of incubation, the MCF-7 nanocapsule uptake was assessed by flow cytometry (Figure 3b). All nanoformulations showed cellular uptake higher than the control group (*p* < 0.05). Additionally, the fluorescence intensity means, considering **f**-LNC^+^ as reference, demonstrated that **f**-Phe-Zn-MLNC-MTX(OEt)_2_, **f**-MTX(OEt)_2_-Zn-MLNC and **f**-MTX-Zn-MLNC-MTX showed significantly higher (*p* < 0.05) nanocapsule cellular uptake than **f**-LNC^+^ (Figure 3c). Furthermore, **f**-MTX(OEt)_2_-Zn-MLNC and **f**-MTX-Zn-MLNC-MTX promoted even higher nanocapsules uptake by MCF-7 cells (*p* < 0.05) compared to **f**-Phe-Zn-MLNC-MTX(OEt)_2_ because of the overexpression of folate receptors on the cellular surface.

Confocal fluorescence microscopy was performed by analyzing MCF-7 cells after 24 h of incubation with the fluorescent-labeled-nanoformulations (supporting information Figure S1). First, the images were recorded without excitation using differential interface contrast (Figure S1, column 1). Next, the images were recorded using a 559 nm laser and a red fluorescence channel (Figure S1, column 2) showing red emission from the PCL-RhoB conjugate (nanocapsule polymeric wall). Nanocapsule cellular uptake by MCF-7 was determined for **f**-LNC^+^, **f**-Phe-Zn-MLNC-MTX(OEt)_2_, **f**-MTX(OEt)_2_-Zn-MLNC and **f**-MTX-Zn-MLNC-MTX formulations after observing the merged images (Figure S1, column 3) by overlapping of channels (Figure S1, columns 1 and 2).

Confocal fluorescence microscopy analysis of MCF-7 incubated with the double-labeled formulation (**ff**-MTX-Zn-MLNC-MTX) was performed using differential interface contrast and two lasers for excitation at 405 nm (5AHBO-C8) and at 559 nm (PCL-RhoB) and the respective channels to record the images (supporting information Figure S2). Cells are shown in Figure S2a, 5AHBO-C8 blue emission in Figure S2b, PCL-RhoB red emission in Figure S2c and the merged image in Figure S2d. The double-labeled **ff**-MTX-Zn-MLNC-MTX uptake by MCF-7 cells was confirmed by observing the magenta secondary color corresponding to the co-localization of blue and red primary colors respectively emitted by 5AHBO-C8 and PCL-RhoB (Figure S2d). In addition, in Figure S2d, we also observed the cellular regions where the blue emission band was exclusively recorded, suggesting the release of 5AHBO-C8 from the nanocapsules within the incubation period of time.

### 2.3. In Vitro Cytotoxicity

The cytotoxicity of the nanoformulations was evaluated using the MTT assay in both immortalized human keratinocyte line (HaCaT) and in human breast carcinoma cell line (MCF-7). Similar surface areas of nanocapsules were applied to compare the nanoformulations and the blank-nanoformulation. Equivalent dose was selected for the treatments corresponding to a nanocapsule surface area of 189 cm^2^·mL^−1^. For the nanoformulations containing methotrexate or its diethyl ester, the applied area (189 cm^2^·mL^−1^) was a dose equivalent to 2.74 mmol of drug (or pro-drug) per liter of formulation. Therefore, for comparative purposes, the MTX and MTX(OEt)_2_ solutions were applied at the same dose (2.74 mmol·L^−1^).

**Figure 4:**
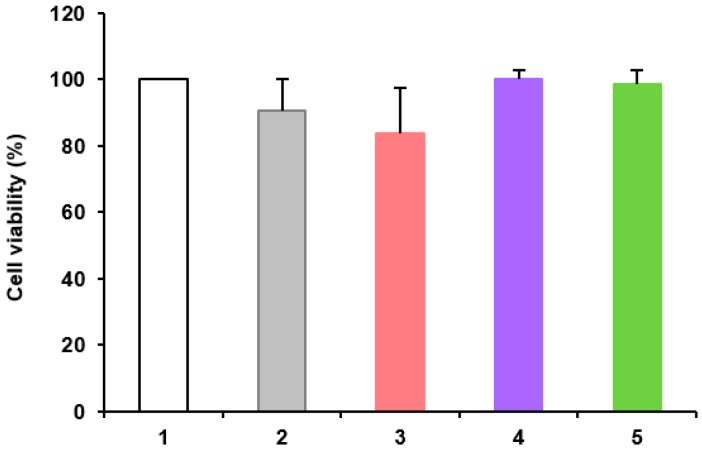
In vitro cytotoxicity in healthy immortalized human keratinocytes (HaCaT) investigated by MTT: (**1**) Control 1 (DMEM); (**2**) LNC^+^; (**3**) Phe-Zn-MLNC-MTX(OEt)_2_; (**4**) MTX(OEt)_2_-Zn-MLNC; (**5**) MTX-Zn-MLNC-MTX. Data are presented as mean ± SD (*n* = 3).No statistical significant difference (*p* > 0.05) was observed between treatments (ANOVA, Tukey).

**Figure 5:**
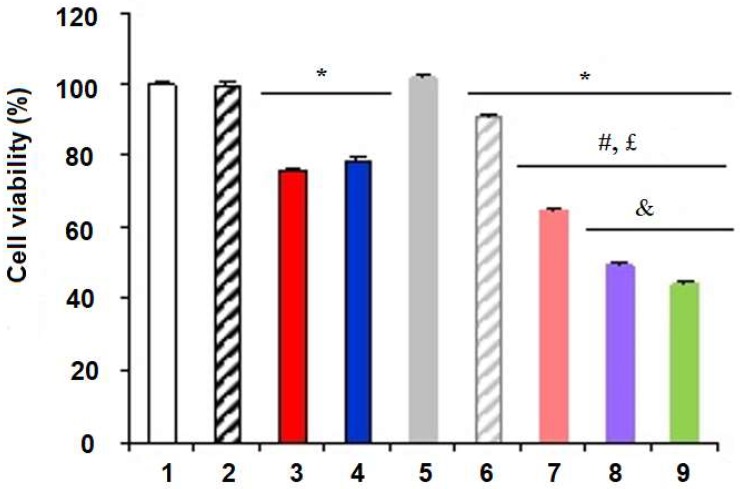
In vitro MCF-7 cells viability determined by MTT test, where (**1**) Control 1 (DMEM); (**2**) Control 2 (0.1% DMSO); (**3**) MTX(OEt)_2_ solution; (**4**) MTX solution; (**5**) LNC^+^ (blank formulation); (**6**) Phe-Zn-MLNC (blank formulation); (**7**) Phe-Zn-MLNC-MTX(OEt)_2_; (**8**) MTX(OEt)_2_-Zn-MLNC; (**9**) MTX-Zn-MLNC-MTX. Data represent the mean ± SD (*n* = 3), * *p* < 0.05 vs. Control (ANOVA, Tukey); # *p* < 0.05 vs. MTX solution (ANOVA, Tukey); £ *p* < 0.05 vs. MTX(OEt)_2_ solution (ANOVA, Tukey); & *p* < 0.05 vs. Phe-Zn-MLNC-MTX(OEt)_2_ (ANOVA, Tukey).

In HaCaT cultures, the nanoformulations Phe-Zn-MLNC-MTX(OEt)_2_, MTX(OEt)_2_-Zn-MLNC and MTX-Zn-MLNC-MTX showed similar cell viability (*p* > 0.05) to Control 1 (DMEM) and the blank-nanoformulation (LNC^+^) (Figure 4), whereas, in MCF-7 cultures, significant reductions (*p* < 0.05) in cell viability were observed when MTX and MTX(OEt)_2_ solutions, as well as Phe-Zn-MLNC, Phe-Zn-MLNC-MTX(OEt)_2_, MTX(OEt)_2_-Zn-MLNC and MTX-Zn-MLNC-MTX were applied (Figure 5). MTX(OEt)_2_-Zn-MLNC and MTX-Zn-MLNC-MTX showed similar (*p* > 0.05) cytotoxic effects but much more pronounced than that observed for Phe-Zn-MLNC-MTX(OEt)_2_ (*p* < 0.05). Similar cell viability was observed for LNC^+^ (blank formulation) and controls (DMEM or 0.1% DMSO aqueous solution) (*p* > 0.05). Reduction of cell viability was significantly higher (*p* < 0.05) for Phe-Zn-MLNC-MTX(OEt)_2_, MTX(OEt)_2_-Zn-MLNC and MTX-Zn-MLNC-MTX compared to either the drug solutions [MTX and MTX(OEt)_2_] or the blank-formulation (Phe-Zn-MLNC) (Figure 5).

**Figure 6:**
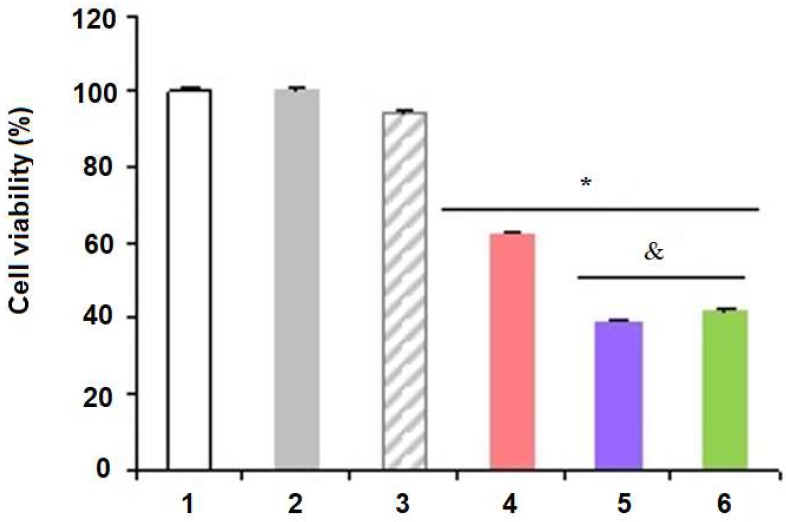
MCF-7 cells viability obtained by cell counting method, where (**1**) Control 1 (DMEM); (**2**) LNC^+^; (**3**) Phe-Zn-MLNC; (**4**) Phe-Zn-MLNC-MTX(OEt)_2_; (**5**) MTX(OEt)_2_-Zn-MLNC; (**6**) MTX-Zn-MLNC-MTX. Data represent mean ± SD (*n* = 3), * *p* < 0.05 vs. Control (ANOVA, Tukey); & *p* < 0.05 vs. Phe-Zn-MLNC-MTX(OET)_2_ (ANOVA, Tukey).

Cell counting was performed using the Trypan blue dye exclusion test. Control 1 (DMEM) and the blank-formulations (LNC^+^ and Phe-Zn-MLNCN) showed similar cell viability (*p* > 0.05) (Figure 6), Phe-Zn-MLNC exhibited similar cell viability (*p* > 0.05) to LNC^+^, showing that both blank-formulations were not cytotoxic. On the other hand, Phe-Zn-MLNC-MTX(OEt)_2_, MTX(OEt)_2_-Zn-MLNC and MTX-Zn-MLNC-MTX significantly decreased (*p* < 0.05) cell viability when compared to Control 1 (DMEM) and to the blank-formulations (LNC^+^ and Phe-Zn-MLNC) (Figure 6). Furthermore, MTX(OEt)_2_-Zn-MLNC and MTX-Zn-MLNC-MTX showed more significant reductions in cell viability (*p* < 0.05) than Phe-Zn-MLNC-MTX(OEt)_2_.

## 3. Discussion

The use of nanostructured materials has been very advantageous in cancer treatment when compared to conventional drug delivery systems [7]. Formulations based on nanotechnology reduce tumor size more effectively than non-encapsulated drugs [8,10,11] due to cellular uptake of drugs. Furthermore, surface functionalization can provide drug to the targeted tissues [13] and reduce cytotoxicity, as demonstrated for mesoporous silica nanoparticles grafted with folic acid intended for two-photon imaging and photodynamic therapy [32].

Previously, we have developed an algorithm to correlate the mechanism of drug encapsulation and drug distribution in nanocapsules dispersed in water [33]. The lipophilicity of the drug, including its acid-base balance (log D), was the physico-chemical parameter with the most profound influence on drug distribution in LNC dispersed in water. In LNC, methotrexate is partially distributed in the PCL-wall of the lipid-core nanocapsules and mainly dissolved in the continuous phase when the interface consisted of PCL and polysorbate 80 [33], whereas methotrexate diethyl ester was distributed in the inner phase (the lipid-core) in the PCL and was partially dissolved in the outer phase (continuous phase). Moreover, the lipid-core nanocapsules were developed using biocompatible and biodegradable materials and can be considered promising building blocks for the development of surface-functionalized nanoparticles. Indeed, we have recently proposed an innovative strategy to bind ligands on the surface of polymeric nanocapsules [23,24].

LNC has a lipid-core surrounded by PCL as a lipophilic architectural component, which was stabilized in water by polysorbate 80-micelles. When lecithin was added to the organic phase, the transmission electron microscopy analysis evidenced four layers of micellar structures coating LNC constituting a hydrophilic corona [34]. Furthermore, interfacial reactions on those lecithin-polysorbate 80-coated LNC, as substrate, produced LNC^+^ and surface-functionalized-MLNC [23]. After adding a chitosan solution to the LNC^−^ formulation, a lipid-core:PCL:chitosanconcentric architecture was obtained by confocal fluorescence microscopy using multi-color fluorescence-labeled MLNC [24].

Here, we have proposed the development of MLNC formulations to perform an in vitro study to evaluate the uptake and antiproliferative activity of methotrexate or its diethyl ester derivative. Phe-Zn-MLNC-MTX(OEt)_2_ and MTX-Zn-MLNC-MTX showed high incorporation efficiency (E%) of methotrexate and its diethyl ester derivative, demonstrating that the chitosan coating, followed by the organometallic complex formation, was able to bind the soluble portion of the drug or its pro-drug to the reactant formulations LNC^−^-MXT and LNC^−^-MTX(OEt)_2_. MTX(OEt)_2_-Zn-MLNC was also developed with high pro-drug incorporation efficiency. We did not oversaturate the colloidal phase since the E% values were higher than 94%. The physico-chemical properties of the formulations were adequate to their use in the in vitro biological evaluations.

Non-tumoral cells, such as HaCaT, have low expression of folate receptors [35]. Activated dihydrofolate reductase increases intracellular levels of tetrahydrofolate triggering the synthesis of purines and pyrimidines, precursors for the synthesis of DNA and RNA for cell replication. On the other hand, tumor cells, in general, such as MCF-7 cell line, grow faster and in a more disordered way and have greater need of nucleotides for DNA and RNA synthesis [36]. The nanocarriers functionalized with folic acid residues showed higher cellular uptake when compared to non-functionalized nanocarriers, such as folate-nanoparticles in cervical cancer cell line (HeLa) [37], folic acid-liposomes in mouth carcinoma line (KB) [38], folate-functionalized paclitaxel-loaded nanoparticles in cervical cancer (HeLa), glioma (C6) in fibroblast cell lines (NIH 3T3) [13] and multifunctional nanoparticles in human breast carcinoma line (MCF-7) and fibroblast cell line (NIH 3T3) [39]. As tumor cells have higher expression of folate receptors on their surface, they capture higher percentages of functionalized nanoparticles. Methotrexate encapsulated in BSA capped gold nanoparticles has effectively increased the drug antiproliferative activity when tested in MCF-7 cell culture [40]. Methotrexate has several adverse effects and its administration in a drug delivery system can be advantageous envisaging an active targeting of tumor tissues and cells. In this way, we hypothesized that MTX-Zn-MLNC-MTX and MTX(OEt)_2_-Zn-MLNC, specific ligands for folate receptor and Phe-Zn-MLNC-MTX(OEt)_2,_ that has phenylalanine on the surface, could exhibit diverse biological behaviors. 

Initially, we have analyzed the influence of the drug localization in the nanocapsule on the cellular uptake using HaCaT and MCF-7 cultures. The HaCaT uptake study conducted with the multiwall lipid-core nanocapsule formulations showed that the nanocapsules were internalized in the cells compared to the control group. In addition, the surface functionalization did not affect the amount of fluorescence emission observed when the drug-containing nanoformulations [Phe-Zn-MLNC-MTX(OEt)_2_, MTX-Zn-MLNC-MTX and MTX(OEt)_2_-Zn-MLNC] were compared to the blank-nanoformulation (LNC^+^). Conversely, in the MCF-7 uptake study, the surface functionalization had an impact on the nanocapsules internalization. Phe-Zn-MLNC-MTX(OEt)_2_, MTX-Zn-MLNC-MTX and MTX(OEt)_2_-Zn-MLNC showed higher cellular uptake than LNC^+^. Moreover, functionalized formulations containing MTX or MTX(OEt)_2_ on their surface [MTX-Zn-MLNC-MTX and MTX(OEt)_2_-Zn-MLNC] exhibited higher uptake by MCF-7 cell line than Phe-Zn-MLNC-MTX(OEt)_2_, in which MTX(OEt)_2_ was encapsulated within the nanocapsules and the surface was functionalized with phenylalanine. In MTX(OEt)_2_-Zn-MLNC and MTX-Zn-MLNC-MTX, methotrexate, a folic acid antagonist or its diester derivative, was functionalizing the surface and the molecules were then more exposed to interact with the folate receptor.

MCF-7 cellular uptake was visualized by confocal fluorescence microscopy using two fluorescent probes, 5AHBO-C8, a benzazole probe (log D = 5) incorporated into the lipid-core [24] and PCL-Rhob, a polymer-dye conjugate forming a blend with PCL [24]. It was possible to infer that the nanocapsules were internalized intact, since magenta secondary light color was observed in the merged image, as well as the blue spots, in the same image, suggesting the release of 5AHBO-C8 from the nanocapsules within 24 h of incubation.

Additionally, even though HaCaT culture showed cell uptake after being incubated with the drug-containing nanoformulations, the cell viability was not affected, demonstrating the safety of the formulations for those non-tumoral cells. MTX(OEt)_2_-Zn-MLNC and MTX-Zn-MLNC-MTX, having MTX or MTX(OEt)_2_ functionalizing the surface of the nanocapsules, showed higher antiproliferative activity than Phe-Zn-MLNC-MTX(OEt)_2_, in which MTX(OEt)_2_ was encapsulated into the lipid-core.

The multiwall lipid-core nanocapsule formulations containing MTX and MTX(OEt)_2_ showed significantly higher antiproliferative activity in breast cancer cells, MCF-7, than the activity observed for the MTX and MTX(OEt)_2_ solutions. Previously, it was demonstrated that glioma strains internalized drug-loaded LNC by endocytosis [41], while formulations containing a specific binder for folate receptor were internalized by endocytosis maybe mediated by the folate receptor [42]. In this way, we suggest that MTX(OEt)_2_-Zn-MLNC and MTX-Zn-MLNC-MTX showed higher cell uptake and increased antiproliferative activity in MCF-7 probably due to the interaction with folate receptors present in those cells. Unequivocally, the formulations showed no cytotoxicity to non-tumoral cells (HaCaT) demonstrating selectivity for the cell lines with increased expression of folate receptors, such as MCF-7 (breast carcinoma cells).

## 4. Materials and Methods

### 4.1. Materials

Poly(ε-caprolactone) (PCL, M_W_ = 14 kg mol^−1^), Span^®^ 60 (sorbitan monostearate, SM), low molecular weight chitosan (CS) (deacetylation degree: 75.6%, viscosity: 20.000 cP), zinc acetate (#383317) and phenylalanine (#P17008) where purchased from Sigma-Aldrich Co. (Saint Louis, MO, USA); Medium chain triglyceride (MCT) and polysorbate 80 (P80) were acquired from Delaware (Porto Alegre, Brazil); Lipoid^®^ S75 (LPS75) (soy phosphatidylcholine, 75% pure) from Lipoid (Ludwigshafen am Rhein, Germany). Methotrexate was acquired from Pharma Nostra (Campinas, Brazil) and dithizone from Merck (Darmstadt, Germany). DMEM (Dulbecco’s modified Eagle’s medium), penicillin/streptomycin, trypsin/EDTA and MTT (3-(4,5-Dimethylthiazol-2-yl)-2,5-diphenyltetrazolium bromide) were supplied by Sigma-Aldrich Co. Fetal Bovine Serum (FBS) was purchased from Gibco (Langley, OK, USA). The fluorescent dye-conjugates 2-(5′-*N*-octylamino-2′-hydroxyphenyl)benzoxazole, 5AHBO-C8 and rhodamine B-poly(ε-caprolactone) conjugate, PCL-RhoB, were prepared and characterized as previously described [24]. All other reagents and solvents used were analytical or pharmaceutical grade and were used as received.

### 4.2. Methods

#### 4.2.1. Preparation of MLNC Containing Methotrexate and Its Ester

##### Methotrexate Ethyl Ester

Initially, the synthesis of methotrexate ethyl ester [MTX(OEt)_2_] was performed according to the methodology previously described [33]. Briefly, methotrexate (1 mmol, 455 mg) was added in ethanol (30 mL) under magnetic stirring at room temperature. Afterwards, 4-dimethylaminopyridine (DMAP; 0.4 mmol, 6 mg) and dicyclohexylcarbodiimide (DCC; 2 mmol, 416 mg) were added. The product was purified by column chromatography using silica gel 60 (70–230 mesh) as stationary phase and chloroform:methanol (99:1 *v*/*v*), using traces of ammonium hydroxide, as eluent.

##### Solutions of Chitosan, Zinc Acetate, Phenylalanine and Methotrexate Ethyl Ester

Aqueous solution of chitosan (1%, *w*/*v*) was prepared by dissolving, in a volumetric flask (10 mL), 101 ± 3 mg of chitosan in 1% acetic acid (*v*/*v*) aqueous solution. Aqueous solution of zinc acetate was obtained by adding 28 ± 1 mg of zinc acetate in a volumetric flask and completing with water to a volume of 10 mL. For phenylalanine solution, 75 ± 2 mg of amino acid was weighed and the volume was completed with water to 10 mL. After complete dissolution, at room temperature, the solutions were filtered using a 0.45 mM membrane (Millipore^®^). The MTX(OEt)_2_ solution was prepared by dissolving 11.3 ± 0.8 mg of the drug in ultrapure water-ethanol mixture (1:1, *v*/*v*) in a volumetric flask (10 mL).

##### Preparation of MTX(OEt)_2_-Zn-MLNC Formulation

The first step of synthesis was to assemble the materials to generate lipid-core nanocapsules stabilized with polysorbate 80-lecithin. LNC^−^ was prepared by adding an ethanol solution of soybean lecithin in a PCL, sorbitan monostearate (SM) and medium chain triglycerides (MCT) acetone solution, at 40 °C. This organic solution was injected in an aqueous phase containing polysorbate 80 (P80) at 40 °C. The turbid solution was stirred for 10 min. The organic solvents were then eliminated and the formulation concentrated to 10 mL in a rotary evaporator (Büchi, Flawil, Switzerland) under reduced pressure at 40 °C. Then, LNC^+^ was produced by adding 1 mL of aqueous solution of chitosan (1%) to 9 mL of LNC^−^ formulation. The reaction was maintained under stirring for 2 h. Then, LNC^+^ (9 mL) was added to 1 mL of zinc acetate solution and (1 min after) of 1 mL of MTX(OEt)_2_ hydroalcoholic solution. This reaction was maintained under stirring for 2 h.

Table 3 shows the comparative quali-quantitative composition of formulations. The final theoretical concentration of methotrexate diethyl ester in the formulation was 2.2 mmol·L^−1^.

##### Preparation of MTX-Zn-MLNC-MTX Formulation

A solution of soybean lecithin 75% in ethanol was added to a colorless transparent acetone solution of PCL, SM, MCT and methotrexate under magnetic stirring at 40 °C. The organic solution was continuously injected into an aqueous solution containing P80 under moderate magnetic stirring at 40 °C (Table 3). The organic solvent was eliminated and part of water removed under reduced pressure at 40 °C, concentrating the formulation to approximately 10 mL. The final volume was adjusted using ultrapure water to 10 mL in a volumetric flask to obtain LNC^−^-MTX. Afterwards, 1 mL of 1% chitosan aqueous solution was slowly added under moderate stirring at 25 °C to 9 mL of LNC^−^-MTX to obtain LNC^+^-MTX. The reaction was maintained under stirring for 2 h. The final step to obtain the functionalized formulations consisted of adding 1 mL of zinc acetate solution and (1 min after) 1 mL of water (to have similar concentrations among formulations) to 9 mL of LNC^+^-MTX under moderate stirring at 25 °C. The reaction was maintained under stirring for 2 h. The final theoretical concentration of methotrexate in the formulation was 2.2 mmol·L^−1^.

##### Preparation of the Phe-Zn-MLNC-MTX(OEt)_2_ Formulation

LNC^−^-MTX(OEt)_2_ was prepared by adding an ethanol solution of soybean lecithin to a transparent and colorless organic phase containing PCL, SM, MCT and methotrexate diethyl ester. Afterwards, the transparent and colorless mixture was continuously injected into an aqueous solution containing polysorbate 80 (P80) under moderate magnetic stirring at 40 °C (Table 3). The organic solvents and part of water were removed under reduced pressure to concentrate the formulation until approximately 10 mL. The final volume was adjusted using ultrapure water to 10 mL in a volumetric flask. Following, 9 mL of LNC^−^-MTX(OEt)_2_ was added to 1 mL of 1% chitosan aqueous solution, which was slowly added under moderate stirring at 25 °C. The reaction was maintained under stirring for 2 h. The final step to obtain the Phe-Zn-MLNC-MTX(OEt)_2_ formulation was performed by adding 1 mL zinc acetate solution and 1 mL of phenylalanine solution to 9 mL of the LNC^+^-MTX(OEt)_2_ turbid solution under moderate stirring at 25 °C. The reaction was maintained under stirring for 2 h. The final theoretical concentration of methotrexate diethyl ester in the formulation was 2.2 mmol·L^−1^. Blank formulations (LNC^+^ and Phe-Zn-MLNC) were also prepared (Table 3).

##### Synthesis of MLNC Formulations Containing Fluorescent Probes

The fluorescent-labeled formulations were prepared with 2.2 mmol·L^−1^ of MTX or MTX(OEt)_2_ according to the procedure described above, substituting 0.5 mg of PCL by 0.5 mg of rhodamine B-poly(ε-caprolactone) conjugate (PCL-RhoB) in the formulations. The fluorescent-labeled formulations were named **f**-MTX(OEt)_2_-Zn-MLNC, **f**-MTX-Zn-MLNC-MTX and **f**-Phe-Zn-MLNC-MTX(OEt)_2_. Fluorescent-labeled-chitosan-coated lipid-core nanocapsule formulation (**f**-LNC^+^) was prepared as control in cellular uptake studies.

Double-fluorescent-labeled formulation was prepared with 2.2 mmol·L^−1^ of MTX (**ff**-MTX-Zn-MLNC-MTX), according to the procedure previously described for multifluorescent-labeled multi-wall lipid-core nanocapsules [24]. Briefly, an acetone solution (25 mL) containing PCL (99.5 mg), PCL-RhoB (0.5 mg), 5AHBO-C8 (1.0 mg), sorbitan monostearate (40 mg) and MCT (120 mg) was added to a soybean lecithin (60 mg) ethanol solution (4 mL). The organic solution was injected into the aqueous solution containing polysorbate 80 (80 mg) under moderate magnetic stirring at 40 °C. The organic solvents and excessive water were removed under reduced pressure at 40 °C to approximately 9 mL using a rotary evaporator. The final volume was adjusted to 10 mL in a volumetric flask.

#### 4.2.2. Physicochemical Characterization of Formulations

##### Laser Diffraction Analysis

Particle size distribution was carried out by laser diffraction (LD) method using a Mastersizer^®^ 2000 (Malvern Instruments Ltd., Malvern, UK) in the size range 0.02 to 2000 µm. Each sample (without any treatment) was inserted into the dispersion unit (Hydro 2000SM-AWM2002, Malvern, UK) containing approximately 100 to 200 mL of distilled water with sufficient amount to achieve obscuration between 1% and 8%. The Malvern software was used to determine the specific area, the mean diameter and polydispersity. Specific surface area was determined considering the density of 1 g m·L^−1^ for all formulations. The diameter distribution curve (expressed by the volume of particles) was used to calculate the equivalent spherical diameter, i.e. volume-weighted mean diameter (D[4,3]) and polydispersity (SPAN), using Equations (1) and (2), respectively.

(1) $$D\left[4,3\right]=\frac{\sum_{i}^{n}{d_{i}^{4}}_{v_{i}}}{\sum_{i}^{n}{d_{i}^{3}}_{v_{i}}}$$

(2) $$\mathrm{SPAN}=\frac{D_{0.9}-D_{0.1}}{D_{0.5}}\times 100$$

where *i* is an index of the population and *d_i_* is the particle diameter of the population *i*, D_0.1_, D_0.5_ and D_0.9_ are the diameters at percentiles 10, 50 and 90 of the particles, respectively, under the cumulative size distribution curve based on the volume of particles.

##### Dynamic Light Scattering

The hydrodynamic diameter calculated by the method of cumulants (Dz-ave) and the polydispersity index (PDI) of the nanoparticles were analyzed by dynamic light scattering (DLS) using a Zetasizer Nano® ZS (Malvern Instruments Ltd., Malvern, UK). To avoid any sample selection, each formulation was diluted (250-folds), without any treatment, in pre-filtered (0.45 μm, Millipore, Bedford, MA, USA) ultrapure water (Milli-Q^®^ water). Diffusion coefficient (DC) was calculated using Stokes-Einstein equation by PCS.

##### Nanoparticle Tracking Analysis

Nanoparticle tracking analysis was performed with a NanoSight LM10 (LM10 & NanoSight NTA 2.0 Analytical Software, NanoSight Ltd., Wiltshire, UK). Brownian motion of nanoparticles [43] and the scattering of the incident radiation (635 nm) were captured by a CCD camera to produce a video. The analysis of each video clip by NTA software 2.0 Build 127 (NanoSight) allowed the measurement of number density of particles and their size distributions. Prior to the analysis, the formulations were diluted 5000× in pre-filtered (0.45 μm, Millipore) ultrapure water (MilliQ^®^). After dilution, the samples were injected into the sample chamber and analyzed for 60 s at room temperature. Gain and shutter were adjusted manually. 

##### Zeta Potential

Zeta potential was determined by means of laser Doppler velocimetry and electrophoretic mobility, utilizing a Malvern Zetasizer^®^ Nano ZS (Malvern Instruments Ltd., Malvern, UK) at 20 °C after dilution of sample (250-folds) in pre-filtered (0.45 μm, Millipore) 10 mmol·L^−1^ NaCl aqueous solution. The results are presented as the average of three replicate analyses.

##### Potentiometry

The pH values of the formulations were measured with a potentiometer (Denver Instrument, Model UB-10, Bohemia, NY, USA) previously calibrated and equipped with an Ag/AgCl reference electrode (Analion V620, Ribeirão Preto, Brazil). Measurements were performed without dilution.

##### Zinc Ion Quantification 

Dialysis of formulations was used to isolate zinc-II soluble in water, as previously described [24]. Formulations were placed in cellulose membranes (10 kDa, Sigma-Aldrich, Saint Louis, MO, USA) and dialyzed against ultrapure water, as medium. Experiments were conducted for 6 h, with moderate agitation at 25 °C. Samples (*n* = 3) were collected every 2 h. Zinc assay was performed using a UV-Vis spectrophotometer (CE UV-1800 PC, PRO-ANÁLISE, Porto Alegre, Brazil) at λ = 514 nm, using dithizone (13 ng·mL^−1^) as a chromogenic agent. Zinc ion concentrations were calculated using Equation (3).

(3) $$C_{\mathrm{Zinc}-\mathrm{II}}=\frac{\left[T-\left(x+y+z\right)\right]}{T}\times 100$$

where *C*_Zinc-II_ is the percentage (%) of Zn^2+^ coordinated nanocapsules (*w*/*v*), *T* is the total concentration of Zn^2+^ added to the formulation and *x*, *y* and *z* are the concentrations of zinc-II in each solution fraction analyzed. To quantify the samples, the method was validated again, considering linearity, precision, repeatability and accuracy.

##### MTX and MTX-Ester Quantifications

Drugs were quantified using an HPLC instrument (Perkin Elmer S-200, Shelton, WA, USA) equipped with a S-200 autosampler and an S-200 UV-VIS detector. The stationary phase consisted of a RP Waters Spherisorb^®^ C_18_ analytical column (150 mm × 4 mm, 4 μm particle size). The formulations containing MTX were extracted with acetonitrile and tetrahydrofuran (THF) 4:6 (*v*/*v*). Meanwhile, formulations containing MTX(OEt)_2_ were extracted with methanol. The dilutions were filtrated prior to analysis (Millipore, 0.45 μm) to separate the dispersed components from the extracted drugs. 

Quantifications of MTX and MTX(OEt)_2_ were performed according to methodologies previously described [33,44]. The injection volume was 20 μL for both analytes. The drugs were detected at 303 nm. For MTX, the mobile phase consisted of metanol:water:tetrahydrofuran (20:70:10 *v*/*v*/*v*), apparent pH of 6.0 ± 0.5 adjusted with 10% (*v*/*v*) acetic acid solution. Flow rate was fixed in 0.8 mL min^−1^. For MTX(OEt)_2_, the mobile phase was methanol:water (80:20 *v*/*v*), with apparent pH of 4.0 ± 0.5 adjusted with 10% (*v*/*v*) acetic acid solution and flow rate of 1.0 mL min^−1^. Both methods showed correlation coefficients higher than 0.99 in the concentration range of 1–50 μg·mL^−1^ and variation coefficients lower than 5% for repeatability and intermediate precision.

To determine the encapsulation (or functionalization) efficiency (EE), 0.3 mL of the formulation was poured into an Ultra-free unit (Microcon, cut-off 10 kDa, Millipore^®^, Burlington, MA, USA) for ultrafiltration-centrifugation at 1844× *g* (RCF) for 5 min in a centrifugal filter device (Sigma^®^1-14, Osterode am Harz, Germany). The encapsulation (functionalization) efficiency (EE%) was calculated using Equation (4).

(4) $$EE\%=\frac{C_{t}-C_{f}}{C_{t}}\times 100$$

where *C_t_* is the total drug concentration in the formulation and *C_f_* is the drug concentration of the ultrafiltrate.

#### 4.2.3. In Vitro Biological Evaluations

##### Cell Cultures

Immortalized Human Keratinocytes (HaCaT) cell line was kindly provided by Luisa L. Villa PhD (ICESP, School of Medicine, University of São Paulo, São Paulo, Brazil) and Silvya S. Maria-Engler PhD (Faculty of Pharmaceutical Sciences, University of São Paulo, São Paulo, Brazil). Human breast cancer cell line (MCF-7) was obtained from American Type Collection Culture (ATCC, Rockville, MD, USA). Both cell lines were cultivated in DMEM (Dulbecco’s Modified Eagle Medium) supplemented with 10% of fetal bovine serum (FBS) at 37 °C and 5% CO_2_. 

##### Cellular Uptake Studies

Cell suspensions were seeded at a density of 6.0 × 10^3^ cells per well in 24-well culture plates. After reaching semi-confluence, HaCaT and MCF-7 cells were incubated with **f**-LNC^+^, **f**-MTX(OEt)_2_-Zn-MLNC, **f**-MTX-Zn-MLNC-MTX and **f**-Phe-Zn-MLNC-MTX(OEt)_2_. The drugs were used at 0.17 mmol·L^−1^ and the cells incubated for 24 h at 37 °C. Afterwards, the media were removed and the wells were washed three times with phosphate-buffered saline (PBS), harvested and analyzed using a flow cytometer (BD FACSVerse) to determine the fluorescence intensity emitted by the polymer-dye conjugate (PCL-RhoB). Relative uptake for each formulation was calculated using the Equation (5), considering **f**-LNC^+^ as control (*FI_i_* = fluorescence intensity of formulation and *FI_f-LNC_^+^* = fluorescence intensity of **f**-LNC^+^):

(5) $$relative\;cellular\;uptake=\frac{FI_{i}}{FI_{f-LNC^{+}}}$$

Qualitative MCF-7 cells uptake was determined by confocal microscopy. Cells were seeded over round glass coverslips placed into 24-well culture plates. After 24 h of incubation at 37 °C with **f**-LNC^+^, **f**-MTX(OEt)_2_-Zn-MLNC, **f**-MTX-Zn-MLNC-MTX and **f**-Phe-Zn-MLNC-MTX(OEt)_2_ (at 0.17 mmol·L^−1^ of each drug), the media were removed, the wells were washed three times with PBS and the coverslips were transferred to the microscope slides. The slides were observed using an Olympus Laser Confocal Microscope (fv1000, Olympus, Tokyo, Japan). PCL-RhoB (red fluorescence) and 5AHBO-C8 (blue fluorescence) were excited using a 559 nm laser and a 405 nm laser, respectively.

##### In Vitro Antiproliferative Activity

Cells were cultivated in 96-well plates and after reaching a semi-confluency, they were treated with aqueous solution of MTX(OEt)_2_ containing 0.1% of DMSO; LNC^+^; Phe-Zn-MLNC; Phe-Zn-MLNC-MTX(OEt)_2_; MTX(OEt)_2_-Zn-MLNC and MTX-Zn-MLNC-MTX, using drug concentrations at 2.74 mmol·L^−1^ and incubated at 37 °C. DMEM supplemented with 10% of FBS was used as control. Cells were also treated with 0.1% of DMSO to determine their toxicity with the same conditions used in the drug derivative solution. 

Negative controls consisted of applying the culture medium (DMEM) or 0.1% DMSO aqueous solution, which was used as solvent for the pure drugs [MTX and MTX(OEt)_2_]. Blank formulations (LNC^+^ and Phe-Zn-MLNC) were used for comparative purposes. Furthermore, the test groups consisted of MTX and MTX(OEt)_2_ solutions and the nanoformulations [Phe-Zn-MLNC-MTX(OEt)_2_, MTX(OEt)_2_-Zn-MLNC and MTX-Zn-MLNC-MTX].

In vitro antiproliferative activity was evaluated using MTT assay (3-(4,5-dimethylthiazol-2-yl)-2,5-diphenyltetrazolium bromide). After 24 h of incubation, media containing the nanoformulations were replaced by a 0.5 mg·mL^−1^ MTT solution. Plates were further incubated at 37 °C for 3 h. Formazan crystals, formed by tetrazolium cleaved in active mitochondria, were dissolved using DMSO and quantified at 570 nm and 630 nm in a microplate reader (Spectramax M2e, SoftMax^®^ Pro Software Interface 5.4.1, Sunnyvale, CA, USA). The results were expressed as percentage compared to controls. Cell viability was considered to be 100%.

Trypan blue dye exclusion test was used to confirm cell viability. After 24 h of treatment application, the medium (DMEM) was removed from the wells and cells were washed with PBS. Trypsin/0.25% EDTA was added to detach cells from the wells. Later on, DMEM/10% FBS was added and cell suspensions were diluted with trypan blue (1:1, *v*/*v*) to selectively stain dead cells. The viable cells (trypan blue negative) were counted with a Neubauer chamber under an optical microscope (Olympus, CX21 model, Tokyo, Japan). Results were expressed as percentage compared to control (DMEM/10% FBS), which represents 100% cell viability.

#### 4.2.4. Statistical Analysis

Data were expressed as mean *±* standard deviation. Results were analyzed by ANOVA followed by Tukey post-test. The statistical analysis was performed using GraphPad Prism 5.0 software (GraphPad Software Inc., La Jolla, CA, USA). The significance level of was set at 5% (*p* ≤ 0.05).

## 5. Conclusions

In this study, we have developed surface-functionalized multi-wall nanocapsules using an innovative strategy based on the use of soft nanocapsules as building blocks. Lipid-core nanocapsules prepared via self-assembly were coated with polysorbate 80-lecithin and reacted with chitosan, zinc acetate and methotrexate, methotrexate diethyl ester or phenylalanine generating three formulations containing different drug binding to the nanocapsules: Phe-Zn-MLNC-MTX(OEt)_2_, MTX-Zn-MLNC-MTX and MTX(OEt)_2_-Zn-MLNC. All formulations were obtained as single nanoscopic populations with no additional purification steps. All formulations showed cell uptake after incubation in immortalized non-tumoral cells (HaCaT) or in tumor cells (MCF-7). However, in MCF-7, the internalization of the nanocapsules was higher for the formulations presenting specific ligands for folate receptor on the surface (MTX(OEt)_2_-Zn-MLNC and MTX-Zn-MLNC-MTX), while in HaCaT, they were similar disregarding the surface of the nanocapsules. No antiproliferative activity was observed in HaCaT culture after incubation with Phe-Zn-MLNC-MTX(OEt)_2_, MTX-Zn-MLNC-MTX and MTX(OEt)_2_-Zn-MLNC, whereas those formulations showed antiproliferative activity against MCF-7 cells. Finally, MTX(OEt)_2_-Zn-MLNC and MTX-Zn-MLNC-MTX which have specific ligands for the folate receptor on the nanocapsule surface, showed enhanced and selective antiproliferative activity to human breast cancer cells (MCF-7). Methotrexate-functionalized-nanocapsules are promising devices for further in vivo preclinical evaluations.

## Figures and Tables

**Table 1:** Physicochemical characterization of LNC^+^, Phe-Zn-MLNC, Phe-Zn-MLNC-MTX(OEt)_2_, MTX(OEt)_2_-Zn-MLNC and MTX-Zn-MLNC-MTX formulations.

	LNC^+^	Phe-Zn-MLNC	Phe-Zn-MLNC-MTX(OEt)_2_	MTX(OEt)_2_-Zn-MLNC	MTX-Zn-MLNC-MTX
D[4,3] (nm)	126 ± 3	125 ± 6	135 ± 10	125 ± 3	131 ± 3
Span	0.9 ± 0.1	1.0 ± 0.2	1.1 ± 0.1	0.8 ± 0.0	1.0 ± 0.1
SA (m^2^ g^−1^)	53 ± 1	56 ± 5	53 ± 2	53 ± 1	52 ± 1
D*_z-ave_* (nm)	130 ± 4	134 ± 10	160 ± 32	148 ± 33	144 ± 9
PDI	0.16 ± 0.03	0.17 ± 0.03	0.13 ± 0.03	0.14 ± 0.04	0.16 ± 0.03
DC (g/L)	(3.6 ± 0.01) × 10^−10^	(3.5 ± 0.02) × 10^−10^	(3.0 ± 0.06) × 10^−10^	(3.2 ± 0.06) × 10^−10^	(3.2 ± 0.02) × 10^−10^
ζ Potential (mV)	+14 ± 3	+18 ± 5	+14 ± 4	+17 ± 5	+18 ± 7
D*_h_* (nm)		143 ± 10	140 ± 9	141 ± 12	176 ± 13
D50 (nm)		133 ± 8	136 ± 12	131 ± 6	160 ± 11
D90 (nm)		185 ± 8	194 ± 6	190 ± 3	243 ± 11
PND (×10^12^ particles mL^−1^)		5.7 ± 0.9	4.7 ± 0.5	5.3 ± 0.8	5.7 ± 0.1
[Zn^2+^] (μg·mL^−1^)	-	76 ± 9	86 ± 2	96 ± 9	99 ± 3
[Drug] (μg·mL^−1^)	-	-	115 ± 4	113 ± 3	101 ± 3
E%	-	-	99 ± 1	97 ± 5	94 ± 6

Note: Data are expressed as mean ± standard deviation. Abbreviations: LNC^+^, lipid-core nanocapsules; Phe-Zn-MLNC multiwall lipid-core nanocapsules; Phe-Zn-MLNC-MTX(OEt)_2_, multiwall methotrexate ester-loaded lipid-core nanocapsules; MTX(OEt)_2_-Zn-MLNC, methotrexate ester-functionalized multiwall lipid-core nanocapsules; MTX-Zn-MLNC-MTX, methotrexate-functionalized methotrexate-loaded multiwall lipid-core nanocapsules; D[4,3], volume-weighted mean diameter; SA, surface area; D*_z-ave_*, hydrodynamic diameter by DLS; PDI, polydispersion index; DC, diffusion coefficient; D*_h_*, hydrodynamic diameter by NTA; D50, median diameter; D90, diameter at 90% under the size distribution curve; PND, particles number density; [Zn^2+^], concentration of zinc-II; [Drug], concentration of methotrexate or methotrexate ester; E%, incorporation efficiency.

**Table 2:** Physicochemical characterization of fluorescent-labeled formulations, **f**-LNC^+^, **f**-Phe-Zn-MLNC-MTX(OEt)_2_, **f**-MTX(OEt)_2_-Zn-MLNC, **f**-MTX-Zn-MLNC-MTX and **ff**-MTX-Zn-MLNC-MTX.

	f-LNC^+^	f-Phe-Zn-MLNC-MTX(OEt)_2_	f-MTX(OEt)_2_-Zn-MLNC	f-MTX-Zn-MLNC-MTX	ff-MTX-Zn-MLNC-MTX
D[4,3] (nm)	173 ± 69	138 ± 12	156 ± 33	145 ± 18	190
Span	1.3 ± 0.5	1.1 ± 0.1	1.2 ± 0.1	1.2 ± 0.1	1.1
SA (m^2^ g^−1^)	49 ± 4	53 ± 2	53 ± 2	52 ± 4	53
_D*z-ave*_ (nm)	153 ± 25	143 ± 15	158 ± 20	150 ± 4	144
PDI	0.21 ± 0.07	0.18 ± 0.01	0.18 ± 0.01	0.19 ± 0.01	0.22
DC (g/L)	(3.1 ± 0.05) × 10^−10^	(3.3 ± 0.03) × 10^−10^	(3.0 ± 0.03) × 10^−10^	(3.0 ± 0.01) × 10^−10^	3.2 × 10^−10^
ζ Potential (mV)	+17 ± 3	+17 ± 1	+20 ± 6	+18 ± 2	+15
[Zn^2+^] (μg·mL^−1^)	-	90 ± 5	95 ± 10	100 ± 3	94
[Drug] (μg·mL^−1^)	-	111 ± 3	112 ± 6	102 ± 5	107
E%	-	100 ± 1	96 ± 4	95 ± 8	96

Note: Data are expressed as mean ± standard deviation, except for **ff**-MTX-Zn-MLNC-MTX. Abbreviations: **f**-LNC^+^, fluorescent-labeled lipid-core nanocapsules; **f**-Phe-Zn-MLNC-MTX(OEt)_2_, fluorescent-labeled multiwall methotrexate ester-loaded lipid-core nanocapsules; **f**-MTX(OEt)_2_-Zn-MLNC, fluorescent-labeled methotrexate ester-functionalized multiwall lipid-core nanocapsules; **f**-MTX-Zn-MLNC-MTX, fluorescent-labeled methotrexate-functionalized methotrexate-loaded multiwall lipid-core nanocapsules; D[4,3], volume-weighted mean diameter; SA, surface area; D*_z-ave_*, hydrodynamic diameter by DLS; PDI, polydispersion index; DC, diffusion coefficient [Zn^2+^], concentration of zinc-II; [Drug], concentration of methotrexate or methotrexate ester; E%, incorporation efficiency.

**Table 3:** Quali-quantitative composition of LNC^+^, Phe-Zn-MLNC, Phe-Zn-MLNC-MTX(OEt)_2_, MTX(OEt)_2_-Zn-MLNC and MTX-Zn-MLNC-MTX formulations prepared in triplicate batches.

	LNC^+^	Phe-Zn-MLNC	MTX(OEt)_2_-Zn-MLNC	MTX-Zn-MLNC-MTX	Phe-Zn-MLNC-MTX(OEt)_2_
PCL (mg)	102 ± 1	101 ± 2	100 ± 2	100 ± 1	99 ± 2
SM (mg)	40 ± 2	42 ± 1	40 ± 3	39 ± 1	41 ± 2
MCT (mL)	0.12	0.12	0.12	0.12	0.12
Acetone (mL)	25	25	25	25	25
LPS75 (mg)	59 ± 1	60 ± 2	60 ± 3	61 ± 1	62 ± 1
Ethanol (mL)	4	4	4	4	4
P80 (mg)	80 ± 2	79 ± 3	80 ± 2	80 ± 2	78 ± 1
Water (mL)	50	50	50	51	50
Chitosan (mg)	1	1	1	1	1
Zinc acetate (mg)	-	2.8 *	2.8 *	2.8 *	2.8 *
Phenylalanine (mg)	-	8	-	-	8
MTX (mg)	-	-	-	1.03 ± 0.6	-
MTX(OEt)_2_ (mg)	-	-	1.13 ± 0.8	-	1.12 ± 0.3
Final volume (mL)	11	11	11	11	11

Note: Data are expressed as mean ± standard deviation. * Equivalent 1 mg zinc-II. Abbreviations: LNC^+^, chitosan-coated lipid-core nanocapsules; Phe-Zn-MLNC, multiwall lipid-core nanocapsules functionalized with phenylalanine; Phe-Zn-MLNC-MTX(OEt)_2_, multiwall lipid-core nanocapsules functionalized with phenylalanine with methotrexate ester encapsulated; MTX(OEt)_2_-Zn-MLNC, multiwall lipid-core nanocapsules functionalized with methotrexate ester; MTX-Zn-MLNC-MTX, multiwall lipid-core nanocapsules functionalized with methotrexate; PCL, poly(ε-caprolactone); SM, sorbitan monostearate; MCT, medium chain triglycerides; LPS75, Soybean Lecithin 75%, P80, polysorbate 80; Zn, zinc; MTX, methotrexate; MTX(OEt)_2_, methotrexate ester.

## Supplementary Materials

The following are available online at http://www.mdpi.com/2079-4991/8/1/24/s1, Figure S1: Confocal laser photomicrographies of MCF-7 cells: column 1 corresponds to images recorded using differential interface contrast, column 2 corresponds to images recorded by using red dye fluorescence channel and laser excitation at 559 nm and column 3 corresponds to the merged images of columns 1 and 2; (a) **f**-LNC^+^; (b) **f**-Phe-MLNC-Zn-MTX(OEt)_2_; (c) **f**-MTX(OEt)_2_-MLNC-Zn and (d) **f**-MTX-MLNC-Zn-MTX, Figure S2: Confocal laser photomicrographies of MCF-7 cells after 24 h of incubation with **ff**-MTX-MLNC-Zn-MTX: (a) image obtained using differential interface contrast; (b) image obtained after excitation at 405 nm using blue fluorescence channel; (c) image obtained after excitation at 559 nm using red fluorescence channel; and (d) merged image using 3 channels (grey: cells, blue: emission from 5AHBO-C8 and red: emission from PCL-RhoB).

## References

[1] Bernardi A., Frozza R.L., Meneghetti A., Hoppe J.B., Battastini A.M., Pohlmann A.R., Guterres S.S., Salbego C.G. (2012). Indomethacin-loaded lipid-core nanocapsules reduce the damage triggered by Aβ1-42 in Alzheimer’s disease models. Int. J. Nanomed..

[2] Vyas D., Castro P., Saadeh Y., Vyas A. (2014). The role of Nanotechnology in gastrointestinal cancer. J. Biomed. Nanotechnol..

[3] Chiang C.-H., Hu S.-H., Liao B.-J., Chang Y.-C., Chen S.-Y. (2014). Enhancement of cancer therapy efficacy by trastuzumab-conjugated and pH-sensitive nanocapsules with the simultaneous encapsulation of hydrophilic and hydrophobic compounds. Nanomedicine.

[4] Hoppe J.B., Coradini K., Frozza R.L., Oliveira C.M., Meneghetti A.B., Bernardi A., Pires E.S., Beck R.C.R., Salbego C.G. (2013). Free and nanoencapsulated curcumin suppress β-amyloid-induced cognitive impairments in rats: Involvement of BDNF and Akt/GSK-3β signaling pathway. Neurobiol. Learn. Mem..

[5] Jaques J.A.S., Doleski P.H., Castilhos L.G., da Rosa M.M., Souza V.C., Carvalho F.B., Marisco P., Thorstenberg M.L., Rezer J.F., Ruchel J.B. (2013). Free and nanoencapsulated curcumin prevents cigarette smoke-induced cognitive impairment and redox imbalance. Neurobiol. Learn. Mem..

[6] Li J., Hu X.L., Liu M., Hou J., Xie Z.G., Huang Y.B., Jing X.B. (2014). Complex of cisplatin with biocompatible poly(ethylene glycol) with pendant carboxyl groups for the effective treatment of liver cancer. J. Appl. Polym. Sci..

[7] Cheng F., Cai W. (2014). Tumor Vasculature Targeting: A Generally Applicable Approach for Functionalized Nanomaterials. Small.

[8] Guo J., Gao X., Su L., Xia H., Gu G., Pang Z., Jiang X., Yao L., Chen J., Chen H. (2011). Aptamer-functionalized PEG-PLGA nanoparticles for enhanced anti-glioma drug delivery. Biomaterials.

[9] Cabral H., Makino J., Matsumoto Y., Mi P., Wu H., Nomoto T., Toh K., Yamada N., Higuchi Y., Konishi S. (2015). Systemic Targeting of Lymph Node Metastasis through the Blood Vascular System by Using Size-Controlled Nanocarriers. ACS Nano.

[10] Nogueira D.R., Tavano L., Mitjans M., Pérez L., Infante M.R., Vinardell M.P. (2013). In vitro antitumor activity of methotrexate via pH-sensitive chitosan nanoparticles. Biomaterials.

[11] Sukthankar P., Avila L.A., Whitaker S.K., Iwamoto T., Morgenstern A., Apostolidis C., Liu K., Hanzlik R.P., Dadachova E., Tomich J.M. (2014). Branched amphiphilic peptide capsules: Cellular uptake and retention of encapsulated solutes. Biochim. Biophys. Acta.

[12] Liang J., Wuc W.-L., Xub X.-D., Zhuo R.-X., Zhang X.-Z. (2014). pH Responsive micelle self-assembled from a new amphiphilic peptide as anti-tumor drug carrier. Colloids Surf. B Biointerfaces.

[13] Wang J., Liu W., Tu Q., Wang J., Song N., Zhang Y., Nie N., Wang J. (2011). Folate-decorated hybrid polymeric nanoparticles for chemically and physically combined paclitaxel loading and targeted delivery. Biomacromolecules.

[14] Couvreur P., Barrat G., Fattal E., Legrand P., Vauthier C. (2002). Nanocapsule Technology: A Review. Crit. Rev. Ther. Drug Carr. Syst..

[15] Hilgenbrink A.R., Low S.P. (2005). Folate-receptor mediated drug targeting: From therapeutics to diagnostics. J. Pharm. Sci..

[16] Guo J., Schlich M., Cryan J.F., O’Driscoll C.M. (2017). Targeted Drug Delivery via Folate Receptors for the Treatment of Brain Cancer: Can the Promise Deliver?. J. Pharm. Sci..

[17] Stella B., Arpicco S., Peracchia M.T., Desmaele D., Hoebeke J., Renoir M., D’Angelo J., Cattel L., Couvreur P. (2000). Design of folic acid-conjugated nanoparticles for drug targeting. J. Pharm. Sci..

[18] Issarachot O., Suksiriworapong J., Takano M., Yumoto R., Junyaprasert V.S. (2014). Folic acid-modified methotrexate-conjugated PEGylated poly(e-caprolactone) nanoparticles for targeted delivery. J. Nanopart. Res..

[19] Wang C., Cheng L., Liu Y., Wang X., Ma X., Deng Z., Li Y., Liu Z. (2013). Imaging-Guided pH-sensitive photodynamic therapy using charge reversible up conversion nanoparticles under near-infrared light. Adv. Funct. Mater..

[20] Mello S.B., Tavares E.R., Bulgarelli A., Bonfa E., Maranhão R.C. (2013). Intra-articular methotrexate associated to lipid nanoemulsions: Anti-inflammatory effect upon antigen-induced arthritis. Int. J. Nanomed..

[21] Kohler N., Sun C., Wang J., Zhang M. (2005). Methotrexate-modified supermagnetica nanoparticles and their intracellular uptake into human cancer cells. Langmuir.

[22] Yurgel V.C., Oliveira C.P., Begnini K.R., Schultze E., Thurow H.S., Leon P.M., Dellagostin O.A., Campos V.F., Beck R.C.R., Guterres S.S. (2014). Methotrexate diethyl ester-loaded lipid-core nanocapsules in aqueous solution increased antineoplastic effects in resistant breast cancer cell line. Int. J. Nanomed..

[23] Bender E.A., Cavalcante M.F., Adorne M.D., Colomé L.M., Guterres S.S., Abdalla D.S.P., Pohlmann A.R. (2014). New strategy to surface functionalization of polymeric nanoparticles: one-pot synthesis of scFv anti-LDL(−)-functionalized nanocapsules. Pharm. Res..

[24] Oliveira C.P., Prado W.A., Lavayen W., Büttenbender S.L., Beckenkamp A., Martins B.S., Lüdtke D.S., Campo L.F., Rodembusch F.S., Buffon A. (2017). Bromelain-functionalized multiple-wall lipid-core nanocapsules: Formulation, chemical structure and antiproliferative effect against human breast cancer cells (MCF-7). Pharm. Res..

[25] Ma J., Jemal A., Ahmad A. (2013). Breast cancer statistics. Breast Cancer Metastasis and Drug Resistance.

[26] Boing A.F., Vargas S.A.L., Boing A.C. (2007). A carga das neoplasias no Brasil: Mortalidade e morbidade hospitalar entre 2002–2004. Rev. Assoc. Med. Bras..

[27] Guiu S., Michiels S., Andre F., Cortes J., Denkert C., Di Leo A., Hennessy B.T., Sorlie T., Sotiriou C., Turner N. (2012). Molecular subclasses of breast cancer: How do we define them? The IMPAKT 2012 Working Group Statement. Ann. Oncol..

[28] Mendonça M.A., Cunha F.Q., Murta E.F., Tavares-Murta B.M. (2006). Failure of neutrophil chemotactic function in breast cancer patients treated with chemotherapy. Cancer Chemother. Pharm..

[29] Shapira A., Livneya Y.D., Broxtermanc H.J., Assaraf Y.G. (2011). Nanomedicine for targeted cancer therapy: Towards the overcoming of drug Resistance. Drug Resist. Updat..

[30] Banerjee D., Mayer-Kuckuk P., Capiaux G., Budak-Alpdogan T., Gorlick R., Bertino J.R. (2002). Novel aspects of resistance to drugs targeted to dihydrofolate reductase and thymidylate synthase. Biochim. Biophys. Acta.

[31] Selga E., Oleaga C., Ramírez S., Almagro M.C., Noé V., Ciudad C.J. (2009). Networking of differentially expressed genes in human cancer cells resistant to methotrexate. Genome Med..

[32] Lebret V., Raehm L., Durand J.-O., Smaïhi M., Werts M.H.V., Blanchard-Desce M., Méthy-Gonnod D., Dubernet C. (2008). Surface functionalization of two-photon dye-doped mesoporous silica nanoparticles with folic acid: Cytotoxicity studies with HeLa and MCF-7 cancer cells. J. Sol-Gel Sci. Technol..

[33] Oliveira C.P., Venturini C.G., Donida B., Poletto F.S., Guterres S.S., Pohlmann A.R. (2013). An algorithm to determine the mechanism of drug distribution in lipid-core nanocapsule formulations. Soft Matter.

[34] Cé R., Marchi J.G., Bergamo V.Z., Fuentefria A.M., Lavayen V., Guterres S.S., Pohlmann A.R. (2016). Chitosan-coated dapsone-loaded lipid-core nanocapsules: Growth inhibition of clinical isolates, multidrug-resistant *Staphylococcus aureus* and *Aspergillus* ssp. Colloids Surf. A Physicochem. Eng. Asp..

[35] Kang M.J., Park S.H., Kang M.H., Park M.J., Choi Y.W. (2013). Folic acid-tethered Pep-1 peptide-conjugated liposomal nanocarrier for enhanced intracellular drug delivery to cancer cells: Conformational characterization and in vitro cellular uptake evaluation. Int. J. Nanomed..

[36] Chintamani, Singhal V., Singh J.P., Lyall A., Saxena S., Bansal A. (2004). Is drug-induced toxicity a good predictor of response to neoadjuvant chemotherapy in patients with breast cancer?—A prospective clinical study. BMC Cancer.

[37] Zhou S., Li Y., Cui F., Jia M., Yang X., Wang Y., Xie L. (2014). Development of multifunctional folate-poly(ethylene glycol)-chitosan-coated Fe_3_O_4_ nanoparticles for biomedical applications. Macromol. Res..

[38] Xiang G., Wu J., Lu Y., Liu Z., Lee R.J. (2008). Synthesis and evaluation of a novel ligand for folate-mediated targeting liposomes. Int. J. Pharm..

[39] Pan J., Liu Y., Feng S.-S. (2010). Multifunctional nanoparticles of biodegradable copolymer blend for cancer diagnosis and treatment. Nanomedicine.

[40] Murawala P., Tirmale A., Shiras A., Prasad B.L.V. (2014). In situ synthesized BSA capped gold nanoparticles: Effective Carrier of anticancer drug methotrexate to MCF-7 breast cancer cells. Mater. Sci. Eng. C Mater. Biol. Appl..

[41] Zanotto-Filho A., Coradini K., Braganhol E., Schröder R., Oliveira C.M., Simões-Pires A., Battastini A.M.O., Pohlmann A.R., Guterres S.S., Forcelini C.M. (2013). Curcumin-loaded lipid-core nanocapsules as a strategy to improve pharmacological efficacy of curcumin in glioma treatment. Eur. J. Pharm. Biopharm..

[42] Bhattacharya R., Patra C.R., Earl A., Wang S., Katarya A., Lu L., Kizhakkedathu J.N., Yaszemski M.J., Greipp P.R., Mukhopadhyay D. (2011). Attaching folic acid on gold nanoparticles using noncovalent interaction via different polyethylene glycol backbones and targeting of cancer cells. Nanomedicine.

[43] Filipe V., Hawe A., Jiskoot W. (2010). Critical Evaluation of Nanoparticle Tracking Analysis (NTA) by NanoSight for the Measurement of Nanoparticles and Protein Aggregates. Pharm. Res..

[44] Sartori T., Seigi Murakami F., Pinheiro Cruz A. (2008). Machado de Campos, A. Development and validation of a fast RP-HPLC method for determination of methotrexate entrapment efficiency in polymeric nanocapsules. J. Chromatogr. Sci..

