# Supplementary material for: Enhanced and Selective Antiproliferative Activity of Methotrexate-Functionalized-Nanocapsules to Human Breast Cancer Cells (MCF-7)
Source: Nanomaterials (Basel). 2018 Jan 4;8(1):24. doi: 10.3390/nano8010024 (PMC5791111; doi:10.3390/nano8010024)
Supplement: Supplementary file 1 [file nanomaterials-08-00024-s001.pdf]

nanomaterials

MDPI

Article

Enhanced and Selective Antiproliferative Activity of Methotrexate-Functionalized-Nanocapsules to Human Breast Cancer Cells (MCF-7)

Catiúscia P. de Oliveira [1], Sabrina L. Büttenbender [2], Willian A. Prado [2], Aline Beckenkamp [1], Ana C. Asbahr [3], Andréia Buffon [1], Silvia S. Guterres [1,3] and Adriana R. Pohlmann [1,2,3,*]

[1] Programa de Pós-Graduação em Ciências Farmacêuticas, Faculdade de Farmácia, Universidade Federal do Rio Grande do Sul, Porto Alegre 90610-000, RS, Brazil; catipadilha@yahoo.com.br (C.P.d.O.); alinee-b@hotmail.com (A.B.); andreia.buffon@ufrgs.br (A.B.); silvia.guterres@ufrgs.br (S.S.G.)

[2] Programa de Pós-Graduação em Química, Departamento de Química Orgânica, Instituto de Química, Universidade Federal do Rio Grande do Sul, Porto Alegre 91501-970, RS, Brazil; sbuttenbender@gmail.com (S.L.B.); will_ap20@hotmail.com (W.A.P.)

[3] Programa de Pós-Graduação em Nanotecnologia Farmacêutica, Universidade Federal do Rio Grande do Sul, Porto Alegre 90610-000, RS, Brazil; acasbahr@hotmail.com (A.C.A.); silvia.guterres@ufrgs.br (S.S.G.)

* Correspondence: adriana.pohlmann@ufrgs.br; Tel.: +55-51-3308-7237

Received: 03 November 2017; Accepted: 28 December 2017; Published: 4 January 2018

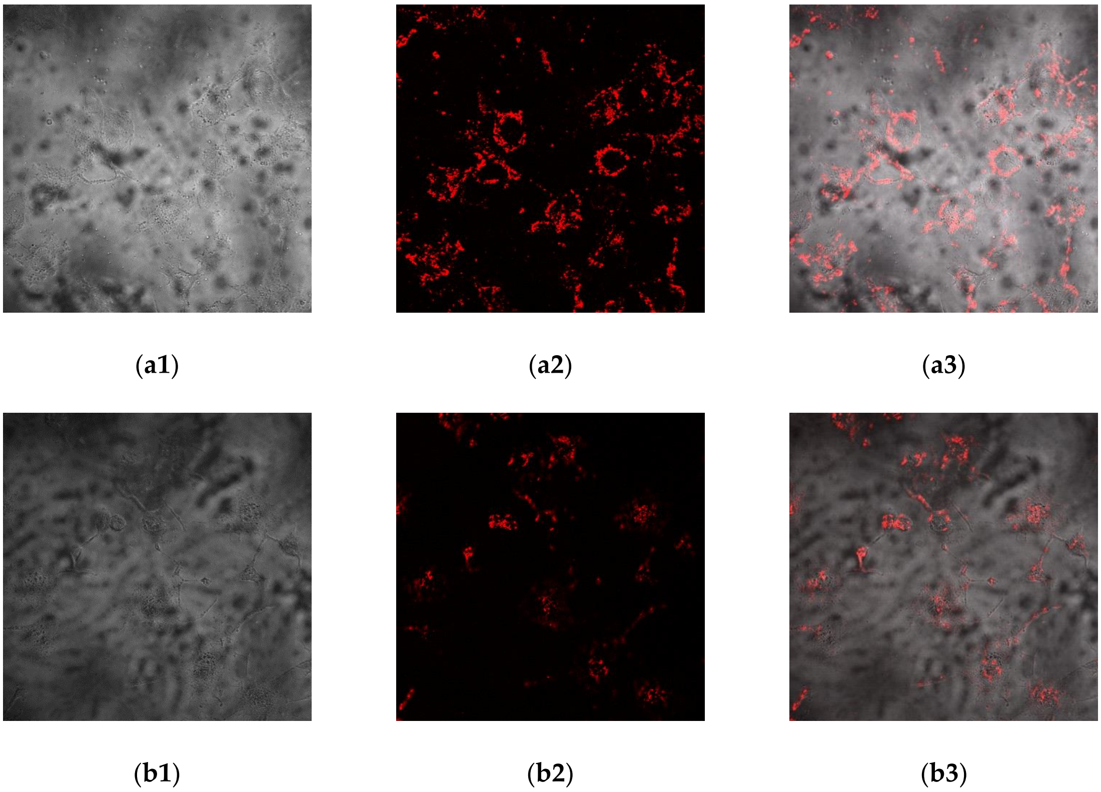

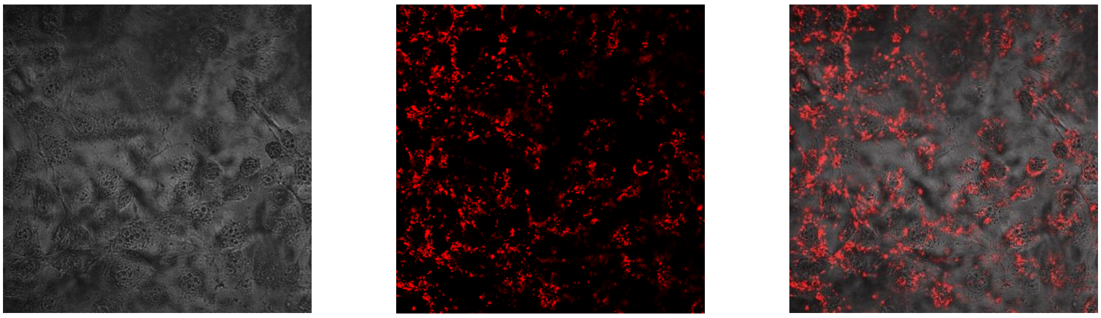

(**c1**) (**c2**) (**c3**)

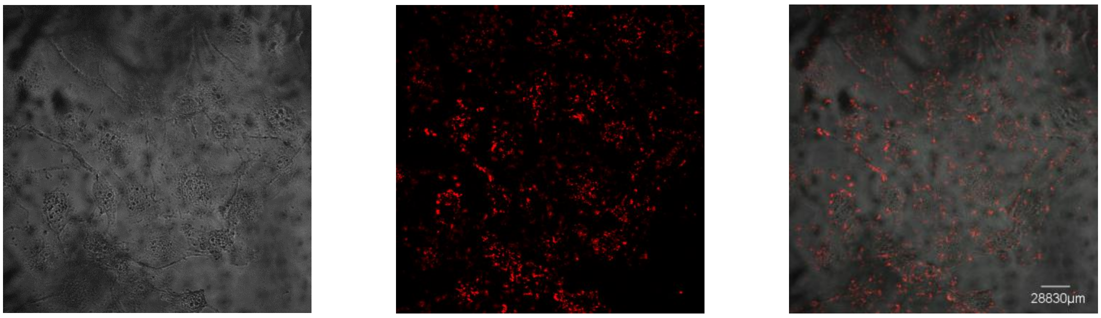

(**d1**) (**d2**) (**d3**)

Figure S1. Confocal laser photomicrographies of MCF-7 cells (bar = 28830 ◎m): column 1 corresponds to images recorded using differential interface contrast, column 2 corresponds to images recorded by using red dye fluorescence channel and laser excitation at 559 nm, and column 3 corresponds to the merged images of columns 1 and 2; (**a**) **f**-LNC$^+$, (**b**) **f**-Phe-MLNC-Zn-MTX(OEt)$_2$, (**c**) **f**-MTX(OEt)$_2$-MLNC-Zn and (**d**) **f**-MTX-MLNC-Zn-MTX.

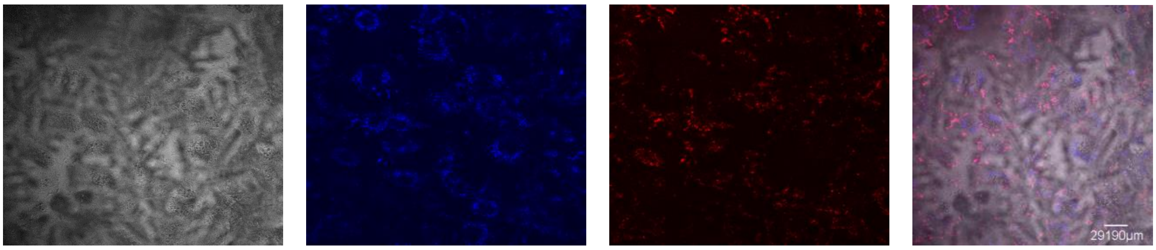

(a) **(b)** **(c)** **(d)**

Figure S2. Confocal laser photomicrographies of MCF-7 cells after 24 h of incubation with **ff**-MTX-MLNC-Zn-MTX: (**a**) image obtain using differential interface contrast; (**b**) image obtain after excitation at 405 nm using blue fluorescence channel; (**c**) image obtained after excitation at 559 nm using red fluorescence channel; and (**d**) merged image using 3 channels (grey: cells, blue: emission from 5AHBO-C8 and red: emission from PCL-RhoB).

© 2017 by the authors. Submitted for possible open access publication under the terms and conditions of the Creative Commons Attribution (CC BY) license (http://creativecommons.org/licenses/by/4.0/).
